# Automatic speech analysis can predict loneliness

**DOI:** 10.1038/s41598-026-45965-5

**Published:** 2026-04-04

**Authors:** Diana Immel, Elisa Mallick, Nicklas Linz, Simon Barton, René Hurlemann, Dirk Scheele

**Affiliations:** 1 Department of Psychiatry and Psychotherapy, School of Medicine and Health Sciences, Carl von Ossietzky University of Oldenburg, Oldenburg, Germany; 2 Karl Jaspers Clinic, Bad Zwischenahn, Germany; 3 ki:elements GmbH, 66111 Saarbrücken, Germany; 4 Research Center One Health Ruhr of the University Alliance Ruhr, Faculty of Medicine, Ruhr University Bochum, 44780 Bochum, Germany; 5 Department of Social Neuroscience, Center of Medical Psychology and Translational Neuroscience, Faculty of Medicine, Ruhr University Bochum, 44780 Bochum, Germany; 6 Department of Psychiatry & Psychotherapy at Karl Jaspers Clinic School of Medicine and Health Science, Carl von Ossietzky University of Oldenburg, Hermann-Ehlers-Str. 7, 26160 Bad Zwischenahn, Germany

**Keywords:** Depression, Loneliness, Speech marker, Social anxiety, Speech analysis, Health care, Psychology, Psychology

## Abstract

Loneliness has been demonstrated to exert a detrimental effect on mental and physical health. It may impede the formation of new social relationships by altering interactional behavior. This study provides a proof-of-concept that loneliness is reflected in altered speech, demonstrating that small yet significant effects can make loneliness audible. Samples of 96 healthy participants (mean age 31.08 years, 53 women) were recorded while they performed a picture description and storytelling task. Paralinguistic markers related to prosodic, formant, source, and temporal qualities of speech were extracted and correlated with loneliness, social anxiety and depression. To validate the diagnostic power, machine learning analyses were conducted for women and men separately. A model comprising all speech features from the picture description task significantly predicted loneliness. However, this model did not predict loneliness from the storytelling task. No single speech feature emerged as a strong predictor of loneliness. A combined model that included both speech features and psychiatric symptoms provided better predictions than psychiatric symptoms alone only in women. Overall, these findings suggest that speech offers a new perspective on how loneliness becomes perceptible to others and how it may disrupt social interactions, thereby fostering chronicity.

**Supplementary Information:**

The online version contains supplementary material available at 10.1038/s41598-026-45965-5.

## Introduction

Human beings are social by nature and are intrinsically motivated to form and maintain interpersonal relationships. When a person’s need to belong is not consistently met, feelings of loneliness develop. Loneliness has a cross-age prevalence rate of up to 33% and has detrimental effects on mental and physical health. It is associated with an increased risk of psychiatric disorders such as depression^1^, psychosis^2^, and social anxiety^3^. Importantly, it is also associated with suicidality^4^ and higher all-cause mortality^5^. Multiple lines of evidence indicate that loneliness is associated with negative cognitive biases^6–8^. For instance, implicit hypervigilance to social threats, increased anticipation of rejection, and negative attributional styles can result in higher expectations of negative social interactions. At the neural level, reduced reactivity and functional connectivity of the anterior insula may reflect impaired integration of trust-related information in lonely individuals^9^. Despite the phenotypic overlap, the neural mechanisms associated with loneliness appear to be distinct from those associated with social anxiety^10^. Importantly, lonely individuals tend to elicit behaviors from interaction partners that lead to the confirmation of their negative expectations, resulting in a self-fulfilling prophecy^6,11^. These cognitive and neural mechanisms are discussed here as theoretical background and help explain why subtle alterations in speech may emerge as markers of loneliness, as communicative behavior both reflects and reinforces social expectations. Consequently, lonely individuals prefer greater social distance in interactions with strangers and benefit less from positive social interactions^9,12^. Meta-analytical evidence also indicates a small negative association between loneliness and prosociality^7^.

Although several studies support the notion that lonely individuals have impaired social interactions, the underlying behavioral mechanisms remain elusive. Preliminary evidence suggests that loneliness is associated with emotion-specific impairments in the recognition of vocal expressions^13^. However, given the bidirectional nature of social interactions, it is conceivable that both altered speech recognition and production may result in dysfunctional social interactions. Previously, we found that the hypothalamic peptide oxytocin facilitates communicative reciprocity by enhancing the salience of vocal expressions^14^. Additionally, we found that interaction-induced oxytocin release is significantly impaired in lonely individuals^9^. These oxytocin-related deficits provide a theoretical basis for why loneliness may be detectable through paralinguistic speech features. Consistent with this, self-reports of communicative competence negatively correlate with loneliness^15^.

Notably, machine learning experiments are increasingly being used to identify speech patterns in patients with mental disorders. Automated speech analysis has been employed to detect apathy in older adults with cognitive impairments and to classify patients with affective disorders^16^, posttraumatic stress disorder^17^, and suicidal ideation^18^. A recent study found that machine learning models based on natural language processing of manually transcribed qualitative interviews can be used to predict loneliness in older adults^19^. However, given the emotion-specific effects of loneliness on speech production, it is still unclear whether changes in speech features associated with loneliness are more pronounced in emotional content and in older individuals, and whether a dyadic setting is required.

To this end, we used the revised UCLA Loneliness Questionnaire (UCLA)^20,21^ to assess loneliness in 96 healthy participants (53 women, mean age 30.85 years; 43 men, mean age 31.37 years). We recorded the participants’ speech during three tasks. They described the Cookie Theft picture from the Boston Diagnostic Aphasia Examination^22^ and were asked to talk about positive and negative life events^23^. Participants were instructed not to recount traumatic events but to select a moderately negative experience. They were further asked to choose a positive event comparable in personal significance and emotional intensity. We used Random Forest regression models to predict the UCLA score from extracted acoustic speech features. Because speech features naturally vary by gender, we conducted the analyses separately for women and men. Due to the phenotypic overlap between loneliness, depression, and social anxiety, we also assessed depression and social anxiety as control variables. We hypothesized that loneliness would be associated with significantly altered paralinguistic markers relating to the prosodic, formant, source, and temporal qualities of speech^24^.

## Results

We observed average loneliness scores of 41.72 in women and 38.02 in men (see Table 1) which are comparable to the normative values reported for the UCLA Loneliness Scale in college students^20^. There were no significant differences in loneliness or depression between women and men (see Table 1). Women reported slightly higher social anxiety scores than men (*U* = 862.50, *p* = .04; see Table 1). In both genders, loneliness significantly correlated with depression (women: *r*_(53)_ = 0.59, *p* < .001; men: *r*_(43)_ = 0.54, *p* < .001) and social anxiety (women: *r*_(53)_ = 0.34, *p* < .005; men: *r*_(43)_ = 0.32, *p* < .05). All questionnaires showed high internal consistency (Cronbach’s Alpha > 0.9; see Table 1).

**Table 1:** Sample description.

	Females *M (SD)*	Males *M (SD)*	*U* (96)	*p*	Cronbach’ s Alpha
Age (years)	30.85 (10.90)	31.37 (9.97)	1246.10	0.43	-
Education (years)	16.28 (2.82)	16.53 (2.42)	1182.50	0.75	-
Loneliness	41.72 (14.71)	38.02 (13.34)	934	0.13	0.96
Depression	6.13 (6.29)	6.98 (7.88)	1186.5	0.73	0.90
Social Anxiety	15.85 (10.86)	12.14 (11.15)	862.50	0.04	0.95

**Fig. 1:**
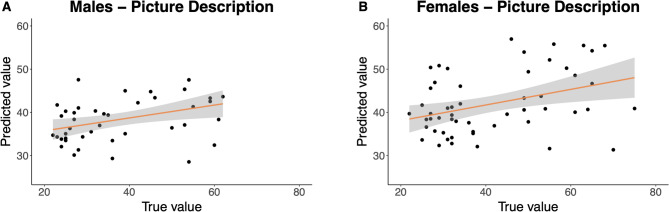
Speech features extracted from the semi-guided picture description task significantly predicted loneliness in males (**A**) and females (**B**). The gray area indicates 95% confidence intervals.

Importantly, the set of speech features extracted from the picture description task by machine learning models significantly predicted loneliness scores of both women and men (see Fig. 1). The model explained 6% and 16% of the variance in women and men, respectively (see Table 2**).**

As expected, depression and social anxiety also predicted loneliness in the machine learning analyses. For women, a combined model that included speech features from the picture description, depression, and social anxiety scores as predictors explained more variance (*R*^*2*^ = 0.21, *p* < .01) than reduced models that included either speech features from the picture description (*R*^*2*^ = 0.06, *p* = .04) or depression and social anxiety scores (*R*^*2*^ = 0.16, *p* = .02). However, in men, the combined model with speech features, depression, and social anxiety scores (*R*^*2*^ = 0.16, *p* = .03) resulted in a worse fit than the reduced model without the picture description speech features (*R*^*2*^ = 0.28, *p* < .01) (see Table 2).

**Table 2:** Results of machine learning experiments for loneliness (picture description).

Features set	Females				Males			
	Random Forest		Randomised Baseline		Random Forest		Randomised Baseline	
	MAE	*R* ^*2*^	*M* (*SD*)	*p*	MAE	*R* ^*2*^	*M* (*SD*)	*p*
Picture Description	11.49	0.06	13.39 (0.95)	**0.04**	10.25	0.16	12.50 (0.92)	**0.02**
Depression + Social Anxiety	11.24	0.16	14.35 (1.48)	**0.02**	8.96	0.28	13.10 (1.47)	**< 0.01**
Picture Description + Depression + Social Anxiety	10.47	0.21	13.36 (0.95)	**< 0.01**	10.10	0.16	12.51 (0.91)	**0.03**
Baseline	13.25				12.08			

*Notes.* MAE: Mean absolute error of random forest regression.

Consistent with the multivariate nature of the machine-learning results, loneliness was not driven by a single dominant speech feature; rather, predictive performance relied on the combined contribution of multiple, individually small effects. For picture description, significant correlations between extracted speech features and loneliness were evident in the temporal and source categories (see Table 3). Specifically, higher loneliness was significantly associated with a lower speech to non-speech ratio in women. Similarly, a higher kurtosis value in the amplitude distribution of the signal from the picture description task was associated with greater loneliness. This suggests that volume intensity was distributed more irregularly among lonely women. In men, greater loneliness significantly correlated with fewer pauses between syllables and a shorter phonation time. Additionally, men with higher loneliness scores had a lower sound-to-noise ratio, reflecting poorer voice quality, and a higher mean pitch.

**Table 3:** TOP 5 highest spearman rank partial correlations between speech features and loneliness, for the picture description.

Speech features	Females				
	Speech ratio	Amplitude kurtosis	Peak frequency	Amplitude mean absolute value	Mean power
Coefficient	− 0.29	0.28	− 0.22	− 0.21	0.13
*p*	**0.03**	**0.04**	0.12	0.14	0.34

Speech features	Males				
	Number of pauses	Total phonation time	Sound to noise ratio	Harmonics to noise ratio	Power spectrum ratio
Coefficient	− 0.44	− 0.43	− 0.37	− 0.25	− 0.21
*p*	**< 0.01**	**< 0.01**	**0.02**	**0.10**	0.17

The set of speech features extracted from the free speech emotional storytelling tasks did not significantly predict loneliness in either positive or negative storytelling for women or men. As expected, the combined models (negative/positive storytelling + depression + social anxiety) explained more variance than the reduced models that examined only storytelling for both genders. However, the combined model only became significant (*R*^2^ = 0.09, *p* = .04) for negative storytelling among women (see Tables S1, S2). A repeated-measures ANOVA comparing the three tasks (picture description, positive storytelling, negative storytelling; Table S3) revealed significant task effects across multiple paralinguistic features. Storytelling elicited more continuous speech, reflected in a higher speech ratio and fewer pauses, compared to the picture description task. In contrast, voice-quality measures such as the harmonics-to-noise ratio were lower during storytelling, consistent with stronger emotional modulation. Additional acoustic parameters, including peak frequency, total phonation time, and several amplitude- and power-related measures, also differed significantly between tasks. These findings indicate systematic contextual differences in speech production across the three task conditions.

## Discussion

In the present study, we examined whether loneliness is reflected in speech features in a heterogeneous sample of young healthy adults. Using a machine learning-based statistical approach, we found that paralinguistic markers extracted from a semi-guided picture description task significantly predicted loneliness in both women and men. Speech features from the temporal and source categories appear to be particularly relevant to this association. Interestingly, a model that included both speech features and depression and social anxiety scores enabled a better prediction than a model only with psychiatric symptoms in women, but not men. However, extraction of speech features from positive and negative free storytelling did not significantly predict loneliness.

Loneliness can affect social interactions in numerous ways. For instance, highly lonely individuals prefer greater distance from an unfamiliar interaction partner^9^, exhibit altered gaze processing^25^, and increased gaze towards their conversation partners’ faces^26^. Sleep-deprived participants have been rated as significantly lonelier and less desirable to interact with^27^. Furthermore, blinded experimenters were able to identify whether they were interacting with a lonely or non-lonely individual^9^. A previous study found that loneliness could be predicted from the content of transcribed speech using natural language processing in older adults^19^. However, our findings suggest that loneliness is also reflected in paralinguistic markers. This highlights an innovative shift from “what is said” to “how it is said.” Consistent with the multifaceted nature of loneliness, the present proof-of-concept study builds on previous approaches of natural language processing by demonstrating that alterations in speech related to loneliness are not limited to linguistic content, but can also be detected in paralinguistic domains (e.g., temporal, source-related, spectral or prosodic speech categories).

Interestingly, speech features extracted from emotional storytelling did not significantly predict loneliness. One possibility is that changes in paralinguistic speech patterns induced by arousal during autobiographical recall obscured loneliness-specific markers. Autobiographical narratives can contain substantial social content, such as interactions, belonging, and rejection^28^. However, since participants responded to open-ended prompts, the degree of social embeddedness likely varied widely among individuals, ranging from highly interpersonal to largely nonsocial events. This variability may have increased heterogeneity in linguistic and paralinguistic patterns, reducing the detectability of loneliness-related signals. Furthermore, participants were instructed to avoid traumatic events and select a moderately negative experience paired with a positive event of comparable personal significance. This restriction may have limited the range of emotional and interpersonal intensity captured by the narrative task, thereby weakening potential associations between paralinguistic markers and loneliness.

Supporting the interpretation that the contexts of the tasks differed meaningfully, our analysis comparing the tasks showed that the storytelling conditions elicited more continuous speech (a higher speech-to-pause ratio) than the picture description task. Meanwhile, voice-quality characteristics (e.g., a lower harmonics-to-noise ratio) differed in the expected direction under stronger emotional modulation. The picture description task requires visual analysis, selection of scene-relevant details, and planning of moment-to-moment descriptions. This process may result in increased planning-related pauses and reduced speech continuity. In contrast, once an event is selected, autobiographical narratives may allow for more fluent output. However, emotional arousal and content variability may introduce additional acoustic variance that can obscure loneliness-specific markers. Thus, null findings in storytelling are unlikely to be explained by reduced verbal engagement but may reflect increased heterogeneity and arousal-related variance in autobiographical speech.

In contrast, the picture description task provides a standardized social scene that may more consistently engage perspective-taking and inferences about others’ intentions, roles, and relationships^29^. These social-cognitive demands may directly activate loneliness-related biases in attention and interpretation^6,8^, which could manifest as paralinguistic speech markers. Future work should quantify the social content of autobiographical narratives (e.g., through manual ratings, such as terms for social processes/belonging, pronoun use, and language for mental states) and test whether social content moderates the associations between acoustic markers and loneliness.

Previously, a reduced oxytocinergic response to semi-guided social interactions was observed in individuals with high loneliness^9^. Reduced oxytocin release may impair the transmission of emotional information in social settings because exogenous (e.g. nasally administered) oxytocin enhanced facial and vocal expression of fear and happiness^14^. These observations highlight that the detectability of loneliness-related speech markers is likely task- and context-dependent. Further research is also needed to investigate whether changes in speech features are related to endocrine function.

Although loneliness is an important risk factor for depression and anxiety, accumulating evidence suggests that it should be considered a distinct construct. In a prospective longitudinal study, loneliness predicted subsequent changes in depressive symptomatology, but not vice versa^30^. Similarly, loneliness exhibits a unique neural profile during cognitive control tasks in patients with major depressive disorder and in healthy controls^31^. Additionally, evidence suggests that lonely and non-lonely individuals experience equal subjective valence when engaging in social situations, as well as exhibit comparable amygdala responses to social decisions and striatal responses to positive social feedback^10^. This pattern of responses stands in stark contrast to the findings for social anxiety^32^. In the present study, loneliness significantly correlated with depression and social anxiety in both women and men. Interestingly, the combined predictive model, which included speech features from the Picture Description Task, as well as depression and social anxiety scores, provided a better model fit for women. For men, however, this model showed a poorer fit than the reduced models, a tendency also evident in the storytelling tasks. Baseline acoustic differences between women and men may influence feature distributions and variance structure and could therefore contribute to the observed gender-specific model performance. However, the present data do not allow mechanistic conclusions; future work should use larger, balanced samples and formally test gender as a moderator. These results suggest that loneliness may follow gender-specific pathways. Prior studies support such differences. Specifically, loneliness has been associated with a more pronounced within-network coupling of the default network in men than in women^33^. An interaction between loneliness and gender was also found following an experimental trauma paradigm: more intrusions were reported by lonely men, but not by lonely women^34^. This effect was accompanied by reduced amygdala habituation to repeated fearful faces and amygdala hyperreactivity during fear conditioning in lonely men. Our results contribute to the existing literature by suggesting that the prediction of loneliness through speech may be more strongly moderated by comorbid symptoms in men than in women. While the driving mechanisms remain unclear, emphasizing gender as a potential moderator is an important direction for future hypothesis-driven research on gender-specific pathways of social communication.

There are several limitations to the current study. First, we recruited healthy individuals with varying levels of loneliness, so it is unclear whether our findings can be generalized to patients with depression or anxiety disorders. Additionally, chronic loneliness is a relatively stable construct with trait-like properties^35^. However, it is likely that the adverse health consequences of loneliness depend on its chronicity^36^. Even brief periods of social isolation can lead to decreased energy levels and increased feelings of fatigue^37^, but situational loneliness seems to drive people toward reconnection, while chronic loneliness seems to drive people away from it^12^. We assessed trait-like loneliness using the established UCLA Loneliness Scale, which does not allow conclusions about the chronicity of the perceived social isolation. Finally, cognitive mechanisms such as negative cognitive biases or altered social expectations were not directly measured; thus, we could not test whether these pathways differ by gender or explain speech–loneliness associations. Future work should assess these constructs explicitly and test moderation by gender.

Taken together, these findings provide the first evidence that loneliness can be predicted by paralinguistic markers that are automatically extracted from semi-guided speech. This mechanism may explain why loneliness can be perceived by others and shed light on a pathway by which loneliness may hinder positive interactions, thereby propagating the maintenance of chronic loneliness. Future research should test these approaches in larger, more diverse samples, including clinical populations, and adopt longitudinal designs that capture loneliness dynamics over time. Furthermore, incorporating speech-based assessments into ecologically valid settings, such as everyday social interactions or digital health platforms, could substantially increase their translational potential.

## Methods

### Participants

Eligibility for the study included the following requirements: Participants had to be between 18 and 65 years of age, speak sufficient German, and not have a psychiatric diagnosis or be taking psychiatric medication. Two sources were used to recruit participants. First, healthy participants were recruited through online advertisements and public notices. Second, pre-stratified healthy participants in a group therapy intervention aimed at reducing loneliness were asked to participate in the study before starting the therapy intervention. A total of 105 participants were included in the study. Nine participants were excluded from the analyses due to missing voice recordings or other missing data. The final sample consisted of *N* = 96 people (53 women and 43 men). The mean age was 30.85 years (± *SD*: 10.90) for women and 31.37 years (± *SD*: 9.97) for men. The study was approved by the Ethics Committee of the University Hospital of Bonn and was conducted according to the principles of the Declaration of Helsinki. The study and data analyses were pre-registered (https://osf.io/buqrj/). Participants were enrolled after providing written informed consent and received monetary compensation at the end of the study.

### Study tasks

The “Cookie Theft” picture from the Boston Diagnostic Aphasia Examination is a well-established method for assessing the expressive language skills of children and adults^38^. One feature of the task is that it elicits mental state language^29^. The picture depicts a familiar domestic scene that requires making assumptions about the mental states of the characters. For instance, the mother is daydreaming and therefore does not notice her children climbing on a stool that is about to fall while they scramble for biscuits. In the free emotional storytelling task, the participants were asked to talk about a negative and a positive event in their lives^23^.

### Questionnaires

In addition to the speech assessment, clinical measures were also collected as part of the investigation. Depressive symptoms were assessed using the German version of the Beck Depression Inventory–II (BDI-II; 21 items rated on a 4-point Likert scale; range 0–63)^39^, originally developed by Beck et al.^40^. Social anxiety was assessed using the German version of the Liebowitz Social Anxiety Scale (LSAS)^41^, originally developed by Liebowitz^42^. Trait-like loneliness was measured using a validated German version of the Revised UCLA Loneliness Scale (Version 2) (UCLA)^21^, originally developed by Russell and colleagues^43^. The UCLA is a 20-item questionnaire rated on a 4-point Likert scale (range: 20–80). Numerous validation studies, primarily in non-clinical undergraduate/student samples, have established loneliness as a distinct psychological construct^44–46^. Psychometric test properties, such as retest reliability and internal consistency, are considered satisfactory^47,48^.

### Procedure

All participants attended one 125-minute study session. The objective of the study and the study procedure were explained. The inclusion and exclusion criteria were explicitly assessed, and written consent was requested before the assessment began. All participants were screened using the Mini-International Neuropsychiatric Interview (MINI)^49^. Then, psychometric questionnaires were administered using Qualtrics software (Provo, USA). Then, the speech assessment was administered on an Apple iPad tablet performed by the ∆elta Clinical app^50,51^. This study was part of a larger study, the results of which are described elsewhere. The speech assessment took approximately five minutes per task and was conducted with an experimenter present. During the speech tasks, the tablet recorded the participants’ speech features.

### Data analysis

The speech data consist of various speech features (see Table S4), that were automatically extracted from the audio signal by the iOS app ∆elta Clinical^51^. These features were extracted separately for picture description, positive storytelling, and negative storytelling. They are grouped into four main categories:

1. *Temporal features* indicate the general rate of speech and measure the proportion of speech (e.g., length and connection of speech segments and the pauses between them). These features reflect the effectiveness of speech production and overlap with prosodic speech characteristics, in the form of fluency and rhythm.
2. *Prosodic features* refer to the long-term dynamics of perceived intonation and speech rhythm. These features demonstrate the overall speech melody adapted to a given situation, thereby indicating prosodic competence in terms of appropriate of speech intonation^52,53^. Prosodic features also measure changes in an individual’s speaking style (e.g., perceived intonation or pitch).
3. *Spectral features* represent the relationship between articulatory movements and changes in vocal tract shape. These features include spectral flow, energy, slope and flatness^54^. Spectral features measure the airborne noise caused by the speech signal and the power of the strongest frequency relative to all others, such as background noise, which can be filtered out to improve speech analysis.
4. *Source features* are important markers of voice quality. They represent the auditory perceptibility of changes in vocal fold vibration and vocal tract shape, outside of pitch, loudness, and phonetics. Source features frequently record information about laryngeal qualities, such as breathing, creaking, hardness, and phonation type^55^.

Due to noise (e.g., background noise) in the processing, the speech features espinola zero crossing metric, mean F0, and average amplitude change demonstrated zero values and were excluded from the analysis.

#### Statistical analysis

The database contains 78 speech features and three clinical scores (UCLA, BDI-II and LSAS) from 105 individuals. To account for gender-specific differences in speech characteristics between women and men (e.g., F0-related measures and aspects of voice quality; see Table S5) due to gender-related anatomical and physiological differences^44,46,56^, the data were analyzed separately by gender to reduce confounding and to examine potentially gender-specific associations with loneliness. QQ plots and Shapiro-Wilk tests revealed that the UCLA, BDI-II and LSAS scores did not follow a normal distribution. The total LSAS score was obtained by summing the anxiety and avoidance subscores. During the data analysis process, it was decided to deviate from the originally planned registration. The variable “loneliness” was used as a continuous variable to mitigate the loss of information. Consequently, the area under the curve calculation, which is used to make predictions, was not conducted. Statistical analysis was performed with Rstudio (version 1.4.1103). Spearman rank correlations were calculated between UCLA, BDI-II and LSAS scores and speech characteristics for both genders. Demographic and psychological variables were compared between men and woman with Mann-Whitney-U-tests. Internal consistency of the three clinical scores (UCLA, BDI-II, and LSAS) was calculated (IBM SPSS Statistics (Version 30)) using Cronbach’s alpha, a statistical method for measuring internal consistency in scales or inventories. We compared the picture description and storytelling conditions across the full set of extracted paralinguistic features using ANOVA (see Table S3). The top five highest Spearman rank partial correlations between speech features and loneliness, across all three tasks and adjusting for different covariates, are presented in Tables S6–S13.

#### Machine learning experiments

Random forest regression models were used to predict the UCLA score based on acoustic speech features extracted from speech tasks, as well as the BDI-II and LSAS scores. The features were normalized using a standard scaler. The models were trained using leave-one-out cross-validation and grid search for hyperparameter tuning. Mean absolute error (MAE) and R-squared are reported as performance measures. The model results were compared with the baseline MAE obtained by predicting the population mean. To calculate the statistical significance of the regression models’ performance, a randomized baseline was used, consisting of training an extra tree model several times with the labels permuted each time.

####  Power analyses

To date, no study has examined the relationship between loneliness and automatically extracted speech features. Therefore, an a priori power analysis was conducted for this project using G*Power 3. This analysis was based on the effect size obtained in a previous study that examined the effects of loneliness on affective responsiveness to a positive social interactions^9^. The results showed that the positive mood change induced by an interaction was significantly reduced in participants with high loneliness (*r*(79) = − 0.25, *p* = .03). To reliably replicate this effect of loneliness (with α = 0.05, and power = 0.80, one-tailed *t*-test), at least 95 participants must be tested. To account for possible dropouts, the plan was to test at least 100 participants (50 women).

## Supplementary Information

Below is the link to the electronic supplementary material.

Supplementary Material 1

Supplementary Material 2

## Data Availability

The data will be provided upon reasonable request.
