# Supplementary material for: Automatic speech analysis can predict loneliness
Source: Sci Rep. 2026 Apr 4;16:11604. doi: 10.1038/s41598-026-45965-5 (PMC13056942; doi:10.1038/s41598-026-45965-5)
Supplement: Supplementary file 1 — Supplementary Material 1 [file 41598_2026_45965_MOESM1_ESM.docx]

**Supporting Information**

The sound of loneliness: Prediction of perceived social isolation using automatic speech analysis

| **Table S1.** Results of machine learning experiments for loneliness (negative story) | | | | | | | |  |
| --- | --- | --- | --- | --- | --- | --- | --- | --- |
|  | **Females** | | | | **Males** | | | |
| Features set | Random Forest | | Randomised Baseline | | Random Forest | | Randomised Baseline | |
|  | MAE | *R^2^* | *M* (*SD*) | *p* | MAE | *R^2^* | *M* (*SD*) | *p* |
| Negative Story | 13.29 | -.11 | 13.34 (0.82) | .46 | 12.06 | -.12 | 12.34 (0.96) | .34 |
| Depression + Social Anxiety | 11.24 | .16 | 14.35 (1.48) | **.02** | 8.96 | .28 | 13.10 (1.47) | **<.01** |
| Negative Story + Depression + Social Anxiety | 11.80 | .09 | 13.40 (0.86) | **.04** | 11.53 | -.02 | 12.36 (0.97) | .16 |
| Baseline | 13.25 |  |  |  | 12.08 |  |  |  |

*Notes.* MAE: Mean absolute error of random forest regression

| **Table S2.** Results of machine learning experiments for loneliness (positive story) | | | | | | | | |  | |
| --- | --- | --- | --- | --- | --- | --- | --- | --- | --- | --- |
|  | **Females** | | | | **Males** | | | | | |
| Features set | Random Forest | | Randomised Baseline | | Random Forest | | Randomised Baseline | | | |
|  | MAE | *R^2^* | *M* (*SD*) | *p* | MAE | *R^2^* | *M* (*SD*) | *p* | |  |
| Positive Story | 14.13 | -.24 | 13.51 (0.89) | .70 | 12.43 | -.14 | 12.40 (0.97) | .48 | |  |
| Depression + Social Anxiety | 11.24 | .16 | 14.35 (1.48) | **.02** | 8.96 | .28 | 13.10 (1.47) | **<.01** | |  |
| Positive Story + Depression + Social Anxiety | 12.26 | .02 | 13.44 (0.89) | .09 | 12.22 | -.12 | 12.46 (0.93) | .32 | | |
| Baseline | 13.25 |  |  |  | 12.08 |  |  |  | | |

*Notes. MAE: Mean absolute error of random forest regression*

| **Table S3.** TOP 5 highest spearman rank partial correlations between speech features and loneliness (corrected for depression) for the picture description | | | | | |
| --- | --- | --- | --- | --- | --- |
|  | **Females** | | | | |
| Speech features | Peak Frequency | Speech ratio | Amplitude mean absolute value | Amplitude kurtosis | Power spectrum ratio |
| Coefficient | -.23 | -.18 | -.17 | .14 | -.13 |
| *p* | .10 | .19 | .24 | .32 | .36 |
|  | **Males** | | | | |
| Speech features | Sound to noise ratio | Number of pauses | Total phonation time | Harmonics to noise ratio | Mean power |
| Coefficient | -.38 | -.31 | -.28 | -.27 | -.14 |
| *p* | **.01** | **< .05** | .07 | .08 | .36 |

| \| **Table S4.** TOP 5 highest spearman rank partial correlations between speech features and loneliness (corrected for depression and social anxiety) for the picture description \| \| \| \| \| \| \| --- \| --- \| --- \| --- \| --- \| --- \| \|  \| **Females** \| \| \| \| \| \| Speech features \| Peak frequency \| Speech Ratio \| Amplitude mean absolute value \| Amplitude kurtosis \| Power spectrum ratio \| \| Coefficient \| -.23 \| -.18 \| -.16 \| .14 \| -.13 \| \| *p* \| .10 \| .20 \| .25 \| .33 \| .36 \| \|  \| **Males** \| \| \| \| \| \| Speech features \| Sound to noise ratio \| Number of pauses \| Total phonation time \| Harmonics to noise ratio \| Mean power \| \| Coefficient \| -.39 \| -.31 \| -.28 \| -.27 \| -.15 \| \| *p* \| **.01** \| .05 \| .08 \| .09 \| .35 \| \|  \| \| \| \| \| \|  \| **Table S5.** TOP 5 highest spearman rank correlations between speech features and loneliness, for the negative story \| \| \| \| \| \| \| --- \| --- \| --- \| --- \| --- \| --- \| \|  \| **Females** \| \| \| \| \| \| Speech features \| Power spectrum ratio \| Amplitude third moment \| Mean power \| Total power \| Amplitude mean absolute value \| \| Coefficient \| -.17 \| .16 \| .15 \| .15 \| .13 \| \| *p* \| .23 \| .24 \| .29 \| .29 \| .34 \| \|  \| **Males** \| \| \| \| \| \| Speech features \| Ampltiude kurtosis \| Harmonics to noise ratio \| Speech ratio \| Amplitude mean absolute value \| Power pectrum ratio \| \| Coefficient \| .39 \| -.23 \| -.19 \| -.18 \| -.17 \| \| *p* \| **.010** \| .13 \| .22 \| .24 \| .28 \|      \| **Table S6.** TOP 5 highest spearman rank partial correlations between speech features and loneliness (corrected for depression), for the negative story \| \| \| \| \| \| \| --- \| --- \| --- \| --- \| --- \| --- \| \|  \| **Females** \| \| \| \| \| \| Speech features \| Power spectrum ratio \| Sound to noise ratio \| Peak frequency \| Total phonation time \| Number of pauses \| \| Coefficient \| -.21 \| -.15 \| -.10 \| -.07 \| .07 \| \| *p* \| .13 \| .28 \| .47 \| .60 \| .63 \| \|  \| **Males** \| \| \| \| \| \| Speech features \| Ampltiude kurtosis \| Harmonics to noise ratio \| Amplitude mean absolute value \| Mean power \| Total power \| \| Coefficient \| .30 \| -.28 \| -.19 \| -.17 \| -.17 \| \| *p* \| .05 \| .07 \| .22 \| .29 \| .29 \|  \| **Table S7.** TOP 5 highest spearman rank partial correlations between speech features and loneliness (corrected for depression and social anxiety), for the negative story \| \| \| \| \| \| \| --- \| --- \| --- \| --- \| --- \| --- \| \|  \| **Females** \| \| \| \| \| \| Speech features \| Power spectrum ratio \| Sound to noise ratio \| Peak frequency \| Total phonation time \| Number of pauses \| \| Coefficient \| -.22 \| -.16 \| -.10 \| -.08 \| .07 \| \| *p* \| .13 \| .29 \| .47 \| .60 \| .63 \| \|  \| **Males** \| \| \| \| \| \| Speech features \| Amplitude kurtosis \| Harmonics to noise ratio \| Amplitude mean absolute value \| Mean power \| Total power \| \| Coefficient \| .30 \| -.28 \| -.20 \| -.18 \| -.18 \| \| *p* \| .05 \| .08 \| .20 \| .26 \| .26 \|  \| **Table S8.** TOP 5 highest spearman rank correlations between speech features and loneliness, for the positive story \| \| \| \| \| \| \| --- \| --- \| --- \| --- \| --- \| --- \| \|  \| **Females** \| \| \| \| \| \| Speech features \| Sound to noise ratio \| Amplitude third moment \| Amplitude mean absolute value \| Mean power \| Total power \| \| Coefficient \| -.21 \| .18 \| .15 \| .14 \| .14 \| \| *p* \| .13 \| .20 \| .27 \| .30 \| .30 \| \|  \| **Males** \| \| \| \| \| \| Speech features \| Harmonics to noise ratio \| Sound to noise ratio \| Total phonation time \| Number of paues \| Maximum amlitude \| \| Coefficient \| -.33 \| -.20 \| .16 \| .14 \| -.14 \| \| *p* \| **.03** \| .20 \| .29 \| .37 \| .38 \|   **Table S9.** TOP 5 highest spearman rank partial correlations between speech features and loneliness (corrected for depression), for the positive story   \|  \| **Females** \| \| \| \| \| \| --- \| --- \| --- \| --- \| --- \| --- \| \| Speech features \| Number of pauses \| Sound to noise ratio \| Power spectrum ratio \| Total phonation time \| Harmonics to noise ratio \| \| Coefficient \| -.12 \| -.12 \| -.12 \| -.09 \| -.07 \| \| *p* \| .40 \| .41 \| .41 \| .52 \| .64 \| \|  \| **Males** \| \| \| \| \| \| Speech features \| Harmonics to noise ratio \| Sound to noise ratio \| Max amplitude \| Total phonation time \| Number of pauses \| \| Coefficient \| -.30 \| -.19 \| -.17 \| .13 \| .12 \| \| *p* \| .05 \| .24 \| .28 \| .41 \| .43 \| |
| --- | --- | --- | --- | --- | --- | --- | --- | --- | --- | --- | --- | --- | --- | --- | --- | --- | --- | --- | --- | --- | --- | --- | --- | --- | --- | --- | --- | --- | --- | --- | --- | --- | --- | --- | --- | --- | --- | --- | --- | --- | --- | --- | --- | --- | --- | --- | --- | --- | --- | --- | --- | --- | --- | --- | --- | --- | --- | --- | --- | --- | --- | --- | --- | --- | --- | --- | --- | --- | --- | --- | --- | --- | --- | --- | --- | --- | --- | --- | --- | --- | --- | --- | --- | --- | --- | --- | --- | --- | --- | --- | --- | --- | --- | --- | --- | --- | --- | --- | --- | --- | --- | --- | --- | --- | --- | --- | --- | --- | --- | --- | --- | --- | --- | --- | --- | --- | --- | --- | --- | --- | --- | --- | --- | --- | --- | --- | --- | --- | --- | --- | --- | --- | --- | --- | --- | --- | --- | --- | --- | --- | --- | --- | --- | --- | --- | --- | --- | --- | --- | --- | --- | --- | --- | --- | --- | --- | --- | --- | --- | --- | --- | --- | --- | --- | --- | --- | --- | --- | --- | --- | --- | --- | --- | --- | --- | --- | --- | --- | --- | --- | --- | --- | --- | --- | --- | --- | --- | --- | --- | --- | --- | --- | --- | --- | --- | --- | --- | --- | --- | --- | --- | --- | --- | --- | --- | --- | --- | --- | --- | --- | --- | --- | --- | --- | --- | --- | --- | --- | --- | --- | --- | --- | --- | --- | --- | --- | --- | --- | --- | --- | --- | --- | --- | --- | --- | --- | --- | --- | --- | --- | --- | --- | --- | --- | --- | --- | --- | --- | --- | --- | --- | --- | --- | --- | --- | --- | --- | --- | --- | --- | --- | --- | --- | --- | --- | --- | --- | --- | --- | --- | --- | --- | --- | --- | --- | --- | --- | --- | --- | --- | --- | --- | --- | --- | --- | --- | --- | --- | --- | --- | --- | --- | --- | --- | --- | --- | --- | --- | --- | --- | --- | --- | --- | --- | --- | --- | --- | --- | --- | --- | --- | --- | --- | --- | --- | --- | --- | --- | --- | --- | --- | --- | --- | --- |

| \| **Table S10.** TOP 5 highest spearman rank partial correlations between speech features and loneliness (corrected for depression and social anxiety), for the positive story \| \| \| \| \| \| \| --- \| --- \| --- \| --- \| --- \| --- \| \|  \| **Females** \| \| \| \| \| \| Speech features \| Sound to noise ratio \| Number of pauses \| Power spectrum ratio \| Total phonation time \| Speech ratio \| \| Coefficient \| -.12 \| -.12 \| -.12 \| -.09 \| -.07 \| \| *p* \| .40 \| .40 \| .42 \| .52 \| .64 \| \|  \| **Males** \| \| \| \| \| \| Speech features \| Harmonics to noise ratio \| Sound to noise ratio \| Max amplitude \| Total phonation time \| Number of pauses \| \| Coefficient \| -.30 \| -.18 \| -.17 \| .14 \| .13 \| \| *p* \| .06 \| .25 \| .29 \| .39 \| .41 \| |
| --- | --- | --- | --- | --- | --- | --- | --- | --- | --- | --- | --- | --- | --- | --- | --- | --- | --- | --- | --- | --- | --- | --- | --- | --- | --- | --- | --- | --- | --- | --- | --- | --- | --- | --- | --- | --- | --- | --- | --- | --- | --- | --- | --- | --- | --- | --- | --- | --- | --- | --- | --- | --- | --- | --- |

| **Table S11.** Overview and explanation of extracted speech features | |  |
| --- | --- | --- |
| **Speech features** | **Explanation** | **Category** |
| Speech ratio | Prosodic feature for verbal fluency from speech to non-speech proportion in the audio | Temporal |
| Speech interval | Speech segments uninterrupted by pauses between syllables to measure speech production efficiency | Temporal |
| Harmonics to noise ratio | Ratio between periodic and non-periodic components of speech which reflects voice quality | Source |
| Sound to noise ratio | Ratio between the power of speech signal and the power of background noise that reflects voice quality | Source |
| Mean F0 | Mean value of the fundamental frequency in voiced parts of the audio. Fundamental frequency. or pitch. quantifies speech signal’s periodic components for speech production assessment | Prosodic |
| Total phonation time | Total time duration of all words across all sentences | Temporal |
| Number of pauses | Total number of pauses longer than a time threshold between the syllables | Temporal |
| Average Mel-frequency cepstrals (MFCC) | Decomposition of Mel-frequency cepstral (MFCC) into a range of spectrum coefficients 1 through 20  MFC represents the short-term power spectrum of a sound | Spectral |
| Deltas | First derivative of the average MFCC values that presents the change in the power spectrum | Spectral |
| Delta deltas | First derivative of the average MFCC values that presents the rate of change in the power spectrum | Spectral |
| Espinola zero crossing metric | Measure for the rate at which speech signal crosses the zero reference and its deviation from the reference | Source |
| Average amplitude change | Variation of the signal amplitude over time | Source |
| Amplitude kurtosis | Kurtosis value for distribution of amplitudes i.e. shows how skewed or centred the distribution is | Source |
| Amplitude mean absolute value | Variation of the signal amplitude over time without change direction | Source |
| Amplitude third moment | Skewness of the signal amplitude over time | Source |
| Amplitude fourth moment | Kurtosis of the signal amplitude over time | Source |
| Max amplitude | Measure for the maximum disturbance of the air caused by the speech signal | Source |
| Peak frequency | Frequency of the maximum power value in signal frequency spectrum | Spectral |
| Power spectrum ratio | Power of the most powerful frequency relative to all other frequencies | Spectral |
| Mean power | Mean power transmitted by the signal | Source |
| Total power | Total power transmitted by the signal | Source |
| Jitter | Jitter is a measure of random perturbation in signal periodicity More representative when examining long vowels. Jitter calculation is based on relative jitter. | Source |

*Notes.* A total of 78 distinctive speech features are grouped into 22 main features
